# AMPK Activation of Apoptotic Markers in Human Breast Cancer Cell Lines with Different *p53* Backgrounds: MCF-7, MDA-MB-231 and T47D Cells

**DOI:** 10.31557/APJCP.2019.20.12.3763

**Published:** 2019

**Authors:** Omar S El-Masry, Barry L Brown, Pauline R M Dobson

**Affiliations:** 1 *Department of Clinical Laboratory Sciences, College of Applied Medical Sciences, Imam Abulrahman Bin Faisal University, 31441, Dammam, Saudi Arabia, *; 2 *Department of Human Metabolism, The Medical School, University of Sheffield, Sheffield, S10 2RX, United Kingdom. *

**Keywords:** AMPK, apoptosis, breast cancer, PARP, cell cycle

## Abstract

**Background:**

Downregulation of AMPK has been established as a major contributor to carcinogenesis in many types of human cancer. We sought to investigate the influence of activated AMPK on apoptotic markers in human breast cancer cells differing in their *p53* status, as well as estrogen receptor status (MCF-7 (*p53*+ and *ER+*), MDA-MB-231 (*p53* mutant and *ER-*) and T47D (*p53* mutant and *ER+*)).

**Methods:**

We examined the effect of AICAR-activated AMPK on PARP cleavage, Bax redistribution, the involvement of intrinsic and extrinsic pathways of apoptosis using selective caspase inhibitors and cell cycle progression and p21 levels.

**Results:**

PARP cleavage occurred to a greater extent in MCF-7 and MDA-MB-231 cells, whereas Bax translocation was slower in MDA-MB-231 cells. Although there were quantitative differences in the effect of caspase inhibitors, it was clear that AMPK activation predominately affected the intrinsic pathway of apoptosis. Although, p21 was increased in all 3 cell types, there were quantitative and time differences. Apoptosis, as measured by fluorimetry, was increased in all three cell types.

**Conclusion:**

The impact of AMPK activation was cell type dependent resulting in differential activation of apoptotic markers, confirming that the genetic background of breast cancer may have an influence on the mode of action of AMPK. Thus, different anti-tumour mechanisms may be elicited depending on the cellular genotype.

## Introduction

AMP-activated protein kinase (AMPK), a ubiquitous metabolic regulator, has emerged as an enzyme having numerous regulatory functions both centrally (Andersson et al., 2004; Anderson et al., 2008; Chikahisa et al., 2009) and peripherally (Fryer et al., 2002; Assifi et al., 2005; Kahn et al., 2005), including control of cell growth and survival (Motoshima et al., 2006; El-Masry et al., 2012; Hardie and Alessi, 2013). AMPK has been reported to be both a biomarker by us and others (Huang et al., 2016; Al-Maghrabi et al., 2017) and a therapeutic target (Cao et al., 2019). In addition, we have shown crosstalk between AMPK and the anti-apoptotic PI3 kinase pathway in breast cancer cells (El-Masry et al., 2015). 

A pro-apoptotic effect of AMPK in cancer cells and a mutual relationship between AMPK and *p53* have been reported (Queiroz et al., 2014). AMPK is a direct upstream kinase of Ser15 in the transactivation loop of *p53* (Jones et al., 2005). In the same context, resveratrol-mediated activation of AMPK has also been reported to mediate Serine 20 phosphorylation of p53 in MCF-7 cells. This action induces apoptosis and sensitizes cells to chemotherapy (Hernandez-Valencia et al., 2018). On the other hand, it is activated downstream of the *p53* transcription targets, sestrin 1 and 2 (Levine et al., 2006; Budanov and Karin, 2008) and the β subunit of AMPK is encoded by a p53 responsive gene (Feng et al., 2007). Thus, AMPK activation may be sufficient to trigger p53 dependent apoptosis, however, the implications of AMPK activation on apoptosis in cells expressing mutant forms of *p53* suggests other mediators (El-Masry et al., 2012). Thus, the relationship between AMPK and *p53*, the so-called guardian of the genome, is still to be clarified particularly in breast cancer. A cell cycle regulatory role of AMPK has also been reported (Dasgupta and Milbrandt, 2009; Queiroz et al., 2014). It is an upstream kinase of Thr198 of the cyclin-dependent kinase inhibitor p27 (Liang et al., 2007). It has also been reported that AICAR-mediated activation of AMPK increases expression of *p21* and *p27* genes (Rattan et al., 2005). It is clear, therefore, that activated AMPK could have a regulatory function in apoptosis as well as in cell cycle progression. We have previously reported that the anti-proliferative effect of AMPK on breast cancer cells was partially mediated by apoptosis and was dependent on the genetic profiles of breast cancer cells (El-Masry et al., 2012). The basis of the current study was to investigate further the effect of AMPK activation on the roles of pathways potentially involved in *p53* dependent and independent cell death. To this end, we have investigated the role of AMPK activation on p21, caspases, PARP cleavage and Bax translocation in breast cancer cells with differing *p53* status. 

## Materials and Methods

Breast cancer cell lines, MCF-7 (*p53+* and *ER+*), MDA-MB-231 (*p53* mutant and* ER-*) and T47D (*p53 *mutant and *ER+*) were purchased from the European Collection of cell cultures (ECACC), Porton Down, UK.

5-aminoimidazole carboxamide ribonucleotide (AICAR), from Sigma Aldrich, Poole, Dorset, UK, was used throughout to stimulate AMPK. Dulbecco’s minimal essential medium (DMEM) and Foetal Calf Serum (FCS) were obtained from Gibco BRL, Paisley, UK. Isoton was from Beckman Coulter UK Ltd. Phosphate buffered saline and Trypsin/EDTA were obtained from Sigma-Aldrich, Irvine, Ayrshire, UK. The Cell titer 96® Aqueous One Solution cell proliferation assay was purchased from Promega, Madison, USA. All caspase inhibitors (caspase 3 (Z-DEVD-FMK), 6 (Z-VEID-FMK), 8 (Z-IETD-FMK) and 9 (Z-LEHD-FMK)) were purchased from AMS Biotechnology (Europe) Ltd, Oxon, UK. Propidium iodide (PI) was from BD Pharminogen™. Rabbit Anti-Bax antibody (human and mouse), rabbit anti-poly (ADP-ribose) polymerase (rabbit anti-PARP) polyclonal antibody and ^p21^ Waf1/Cip1 rabbit monoclonal antibody and polyclonal anti-rabbit IgG antibody linked to horseradish peroxidase were all from Cell Signalling Technology^®^, Danvers, MA, USA. Goat Anti-rabbit IgG-FITC antibody was from Santa Cruz Biotechnology, USA. T25 and T75 tissue culture flasks were from Nunc™, Denmark. Tissue culture sterile 6, 24 and 96 well plates were from COSTAR®, USA and sterile tissue culture Petri dishes were from Thermo Scientific (UK Ltd). Lab-tek chamber slides were from Nunc™, New York, USA.

*Methods*

All cells were grown in DMEM plus FCS (10%). 

*Detection of PARP cleavage and the level of total p21 by Western blotting*

1×10^6^ cells were seeded in appropriate tissue culture plates and incubated at 37 ͦ C and 5% CO_2_ in a humidified atmosphere. Cells were then incubated with AICAR (0.83mM) or vehicle (DMEM) for the times indicated in the figure legends. Cell lysates from control and treated cells were collected, followed by protein assay. For Western blotting, equal protein concentrations of control and treated cells (w/v) were premixed with Laemmli sample buffer (BioRad) containing β-mercaptoethanol (20:1) and loaded into wells of pre-formed tris-HCL-SDS gels (BioRad). Proteins were then blotted onto a poly vinyl difluoride (PVDF) membrane followed by polyacrylamide gel electrophoresis (PAGE). The membrane was then blocked overnight in 10% dry milk before being incubated overnight at 4^o^C with rabbit anti-PARP polyclonal antibody or p21 Waf1/Cip1 rabbit monoclonal antibody. The membrane was then washed in TBST four times (15 minutes each) then incubated with polyclonal anti-rabbit IgG antibody (horseradish peroxidase (HRP) linked, then washed again four times before generating bands on X-ray films.

*Effect of AICAR on Bax redistribution*

Cells were sub-cultured, counted using a Coulter counter and 5x10^4^ cells were then seeded into the chambers of lab-tek chamber slides before being incubated overnight at 37°C and 5% CO_2_ to adhere. Adherent cells were then incubated with AICAR (0.83mM) or DMEM for 24 or 72 hours under the same culture conditions. Slides were then washed twice by immersing them in PBS. Cells were then fixed and permeabilized in acetone for 5 minutes at room temperature. Fixed cells were then washed three times in PBS. After removal of excess PBS, cells were incubated with appropriately diluted (1:250) primary rabbit anti-Bax antibody for 30 minutes under humid conditions in the dark. Slides were then washed again as above. Cells were then incubated with goat anti-rabbit IgG-FITC antibody for 30 minutes under the same conditions as for the primary antibody. The wash was repeated, the excess PBS removed and the plastic chamber compartment was then removed according to the manufacturer’s instructions. A Pasteur pipette was then used to remove the remaining PBS. A small drop of the mounting medium suitable for fluorescence studies was then added before applying the cover slip, sealing it and examining slides for Bax localisation under the fluorescence microscope. 

*Co-incubation with selective caspase inhibitors and AICAR on cell proliferation*

Cells were sub-cultured, counted, and 1x10^4^ cells were seeded/well in tissue culture 96 well plates in triplicate sets. Plates were then incubated overnight at 37°C and 5% CO_2_ to equilibrate and adhere. On the next day, caspase inhibitors were diluted 10 times in DMEM in sterile tubes. The final concentration of all caspase inhibitors was made to 20μM and plates were then incubated for one hour prior the addition of AICAR. Controls were treated with 50μl of DMEM. Another set of wells was treated with AICAR in medium. Plates were then incubated for 72 hours in the same culture conditions before adding 20μl of tetrazolium dye to each well, followed by an incubation period of 1-2 hours to assess cell proliferation. Plates were then read using a microplate reader at dual wavelength (490 and 630nm). Results, being means of 3 independent experiments, are presented as histograms.

*Cell cycle analysis*

1×10^6^ cells were seeded in 100mm sterile tissue culture Petri dishes, then incubated overnight at 37^o^C and 5% CO2 to adhere. Cells treated with AICAR (0.83 mM) were incubated with their corresponding controls (medium alone) at 37^o^C and 5% CO_2_ for 24, 48 or 72 hours. Zero time cells were equilibrated for 2 hours in the standard culture conditions. “Zero time” is a term applied to the stage when cells (that are not synchronised) are sub-cultured and plated into dishes and have sufficient time for cells to adhere to the substratum (checked by microscopic viewing), albeit they are rounded in culture at this very early stage before the beginning of the experiment. 

All cells were washed with PBS, and released by being incubated with trypsin/EDTA for 5 minutes at 37^o^C and 5% CO_2_, and then combined with the non-adherent cells. Cells were then washed twice by being spun in ice cold PBS for 10 minutes at 200g and 4^o^C. Cells were then permeabilized in 70% ethanol (1ml) overnight at 4^o^C. The permeabilized cells were then washed twice and 300µl of the DNA staining flurochrome, propidium iodide liquid, was then added. Labelled cells were then incubated overnight at 4^o^C. Forward and side scatter (FSC and SCC) plots were created based on cell size and granularity and the output of the emission spectra of PI (read at FL3H) was plotted as histograms. 

*Statistical analysis*

Statistical comparisons have been done using Graphpad Prism (Version 7). Comparisons between two groups were done using the un-paired student t-test or as indicated otherwise, while analysis of variance (ANOVA) was used for multiple comparisons, unless otherwise indicated for the non-parametric statistics. The difference was considered statistically significant when p values ≤ 0.05. Statistical analysis was applied where appropriate as validated by international standards in the use of established clonal cell lines. We were unable, however, to apply this to Bax translocation that is defined by photographic evidence.

## Results

*AMPK activation resulted in differential PARP cleavage in breast cancer cells*

**Figure 1:**
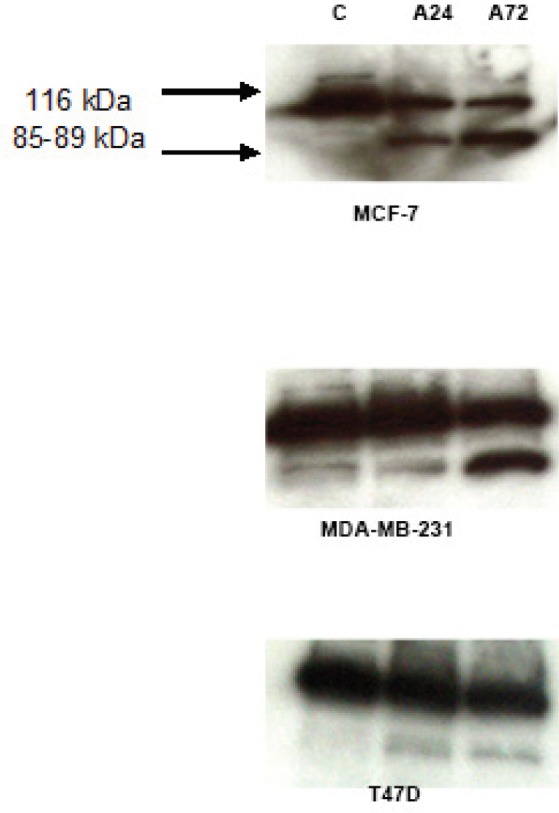
Differential Time-Course of PARP Cleavage was Induced by the AMPK Activator, AICAR. Cells were incubated for 24 or 72 hours with AICAR (0.83uM) and PARP cleavage was assessed by Western blotting. A24 = AICAR 24 hours and A72 = AICAR 72 hours. n=3 independent experiments

Incubation of MCF-7, MDA-MB-231, and T47D cells with AICAR for 24 or 72 hours resulted in an apparent cleavage of PARP enzyme in comparison to the corresponding control cells. This cleavage was more obvious in MCF-7 cells after 24 hours of incubation than in MDA-MB-231 cells where cleavage was marked after 72 hours. Little PARP cleavage was observed in T47D cells (Figure 1).

*Bax redistribution was induced by AICAR-mediated AMPK activation*

**Figure 2:**
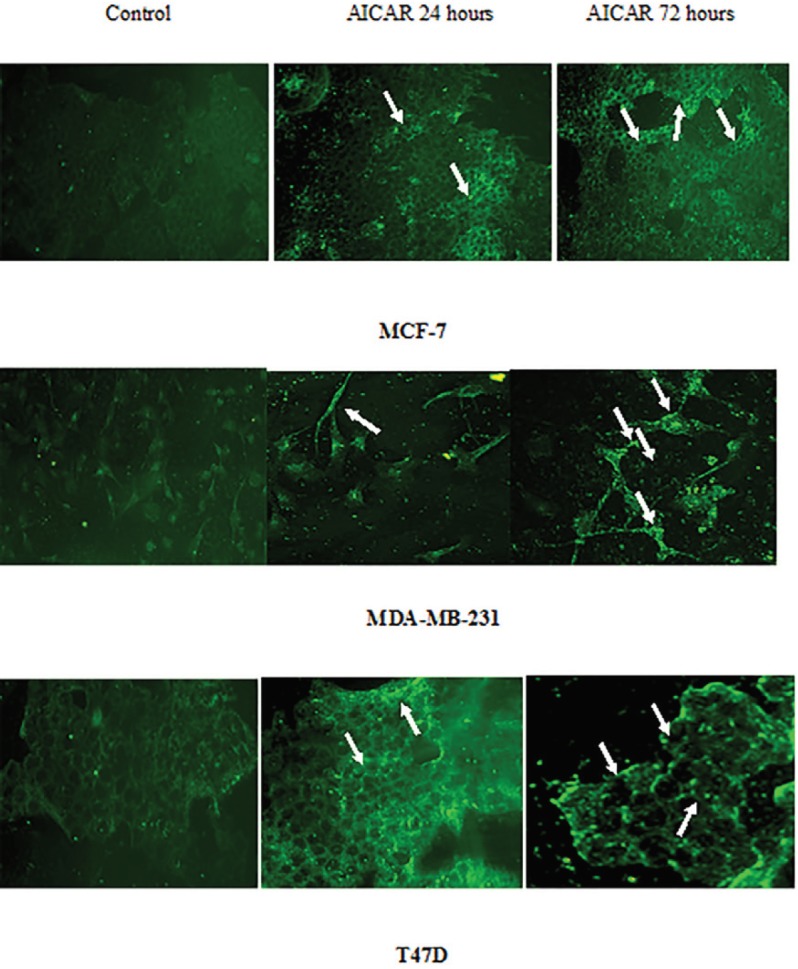
Time-Course of Bax Translocation to Mitochondria in Response to AICAR. Treatment of cells with AICAR resulted in a time dependent translocation of cytosolic Bax to the outer mitochondrial membrane (Punctate pattern; white arrows) detected by fluorescent microscopy. The presence of Bax in cytosol is indicated by the diffused fluorescence pattern in control cells. Three independent fields were assessed in each case

We have previously reported that activated AMPK resulted in disruption of the mitochondrial membrane potential in MCF-7, MDA-MB-231 and T47D cells (El-Masry et al., 2012). The possibility that this effect might be mediated by induction of Bax translocation from cytoplasm to the outer mitochondrial membrane following AMPK activation was investigated here. Immunoanalysis showed that incubation of cells with AICAR (0.83 mM) for 24 and 72 hours resulted in a clear Bax translocation pattern in MCF-7 and T47D cells after 24 hours of incubation. Translocation of Bax was increased after 72 hours of incubation, particularly in MDA-MB-231 and T47D cells (Figure 2). 

*AICAR triggered the intrinsic pathway of apoptosis*

**Figure 3:**
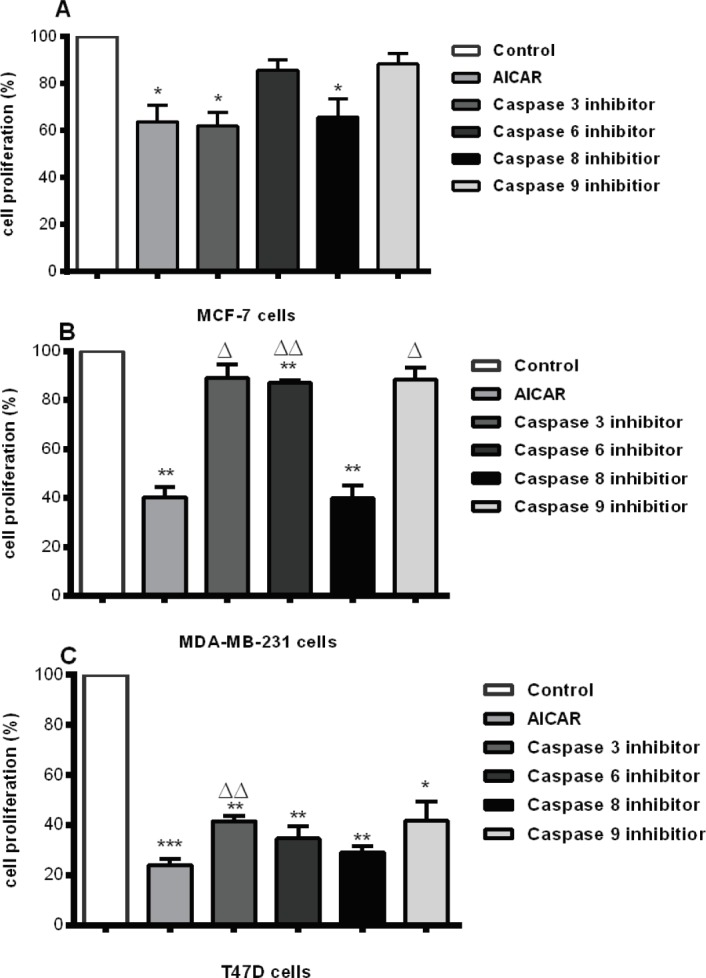
Irreversible Caspase Inhibition Revealed the Involvement of Intrinsic Mitochondrial Apoptosis in the Anti-Proliferative Action of AICAR. Cells were subjected to AICAR in the presence and absence of specific inhibitors of Caspases 3, 6, 8 or 9. AICAR alone was compared to Medium alone (*). The Caspase inhibitors in the presence of AICAR were compared to both Medium alone (*) and to that containing AICAR alone (∆). (* and ∆ = p ≤ 0.05; ** and ∆ = p ≤ 0.01; *** and ∆∆∆ = p ≤ 0.001). n=3 independent experiments of which each points were done in triplicate in each experiment

Co-treatment of MCF-7, MDA-MB-231 and T47D cells with AICAR (0.83mM) and selective irreversible inhibitors of caspase 3, 6, 8 or 9 were compared to cells treated with medium alone or AICAR alone. AICAR caused a significant decrease in proliferation in MCF-7 cells. The responses in MDA-MB-231 cells and T47D cells however was markedly greater (MCF-7 < MDA-MB-231 < T47D) (Figure 3). When caspase inhibitors were added prior to and during incubation with AICAR results, compared to medium alone, showed that caspase 3 and 8 inhibitors were without effect in MCF-7 cells, whilst caspase 6 and 9 returned levels to basal levels of medium alone. In MCF-7 cells, no difference was observed between AICAR alone or in the presence of any of the caspase inhibitors (Figure 3A). In summary, inhibitors of caspases 3 and 8 could not overcome the effects of AICAR, whereas inhibitors of caspases 6 and 9 could.

In MDA-MB-231 cells, the outcome of the effect of caspase inhibitors in the presence of AICAR differed markedly (Figure 3B). Whilst AICAR alone reduced the proliferative rate, recovery by prior and co-incubation with the caspase 3 inhibitor overcame this. Otherwise, the effects of AICAR alone were overcome by caspase inhibitors 6 and 9, but as with MCF-7 cells, caspase 8 inhibition was ineffectual.

In T47D cells, a very marked reduction in cell proliferation was observed in response to AICAR alone (T47D > MDA-MB-231 >> MCF-7 cells) (Figure 3C). In this case, caspase inhibitor 3 partially overcame the effects of AICAR alone. Caspase 3, therefore, cannot account, in total, for the inhibitory action of AICAR; another mechanism is in place in T47D cells. Also, the other caspase inhibitors were unable to overcome the effect of AICAR, nor could they approach the level of proliferation in response to medium alone.

From the above, we can deduce that caspase 8 is probably ineffectual in the actions of AMPK in all three breast cancer cell types. In addition, however, the mechanisms through which AMPK elicits its response differs across the three cell lines in terms of involvement of caspases to accomplish the endpoint. 

*AICAR caused a cell type-dependent increase in p21 level*

**Figure 4:**
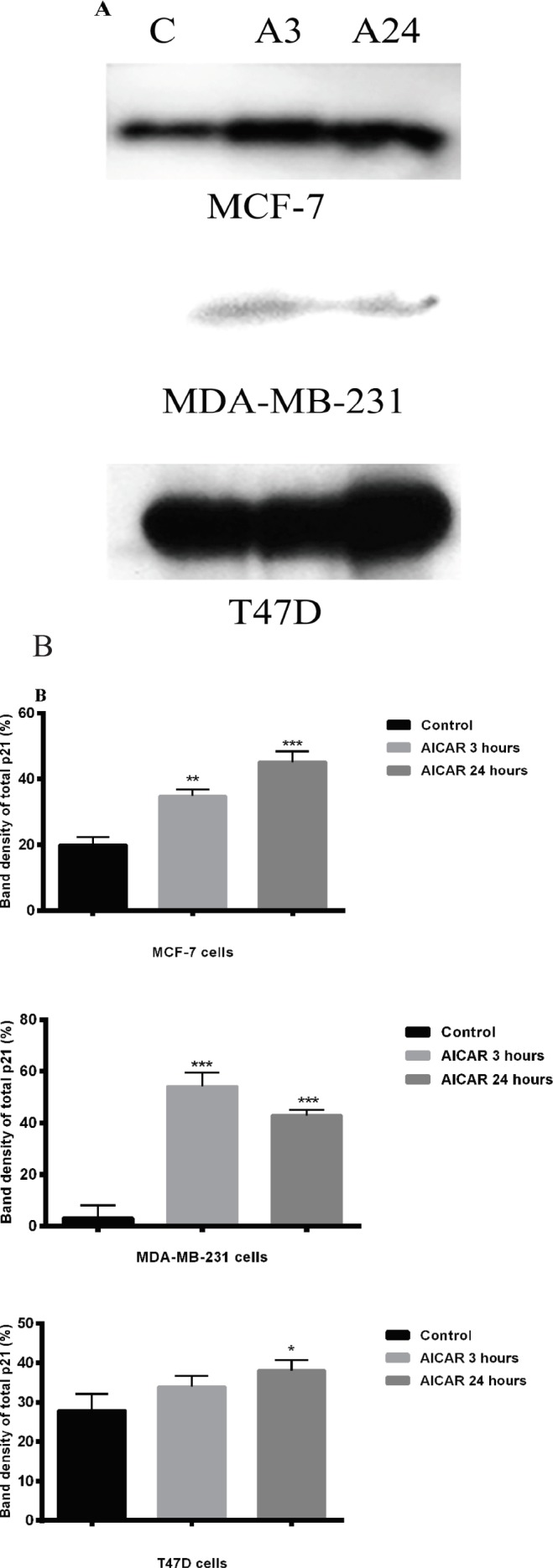
The Effect of AICAR on p21. The p21 status and response to AICAR was investigated in all 3 cell types by Western blotting. Results are presented as blots (A) and as histograms as a result of densitometric scanning (B); n=3 independent experiments. Densitometric scanning permitted the application of statistical analysis of the scans. In each case; *= p < 0.05, ** = p < 0.01, *** = p < 0.001

A significant increase in p21 levels was observed in MCF-7 cells after 3 or 24 hours of incubation with AICAR when compared with the corresponding control cells. A lower level of p21 was hardly detected in MDA-MB-231 cells, however, following incubation with AICAR either for 3 or 24 hours (Figure 4 A and B): a marked increase in the response to AICAR in p21 was noted nonetheless. This was greater than in either of the other 2 cell types, compared to their respective basal levels. In T47D cells, the basal level of p21 was higher and there was a small, but significant, increase in the p21 level after incubation with AICAR for 24 hours, being substantially lower activation compared to the other cell types. This suggested a largely ineffective level of p21 in this cell type despite its greater expression.

In summary, the most striking discovery was that p21 was hardly detectable in MDA-MB-231 cells. All three cell types achieved significant increases in p21 after AICAR exposure. However, remarkably, the cell type with the lowest expression of p21 achieved the highest response. In T47D cells, activation was lesser and slower.

*Effect of AICAR on the sub-G1 phase of the cell cycle*

Flowcytometry results showed that the majority of MCF-7, MDA-MB-231 and T47D cells were in the G1 phase at zero time (results not shown). 

No difference was observed between zero time and the basal levels up to 72 hrs in any cell type. A significant apoptotic response was observed in the MCF-7 cell line after 72 hours of incubation with AICAR. Also, a significant increase in the number of cells in the sub-G1 phase was observed in the MDA-MB-231 cell type after 48 and 72 hours of incubation with AICAR when compared to the corresponding controls. Finally, in T47D cells, there was a significant increase in the number of sub-G1 cells after 48 and 72 hours when compared to the corresponding control cells. These results show that there is little difference between the ultimate responses of the cells from differing genetic backgrounds; they all go into apoptosis after AICAR activated AMPK despite being achieved by different means.

**Figure 5:**
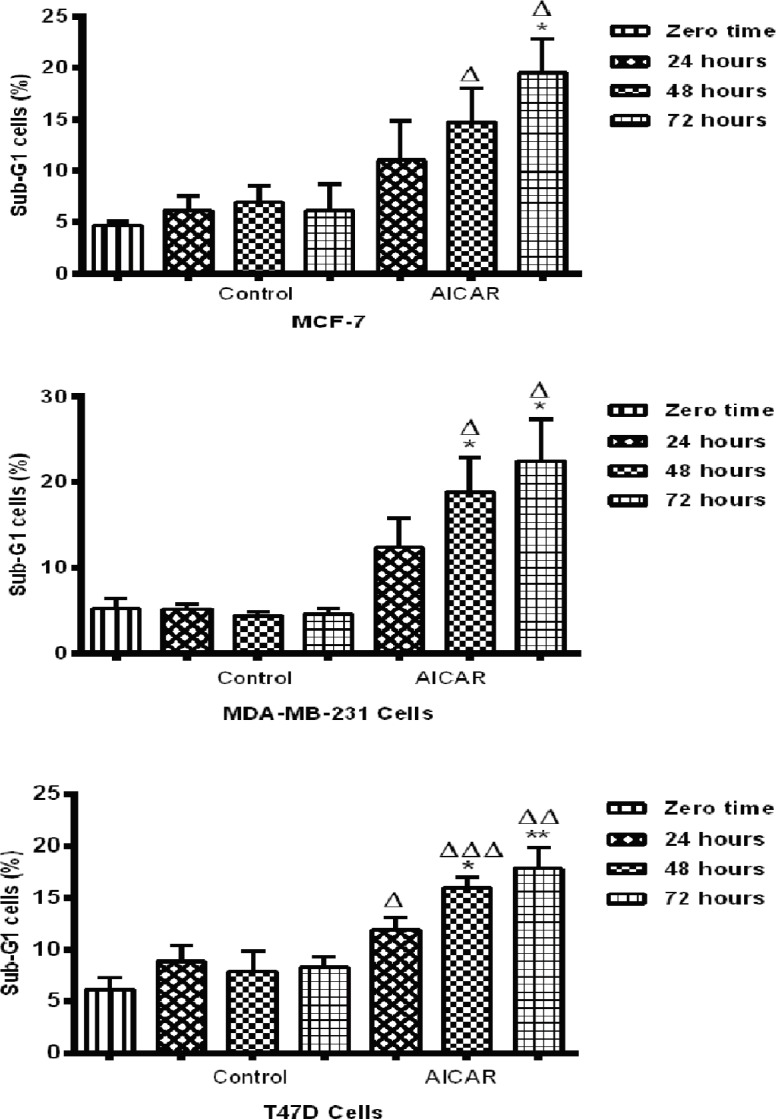
The Effect of AICAR on the Sub G1 Phase of Cell Cycle. AICAR was incubated with each cell type for the times shown. Treated cells were prepared for cell cycle analysis using PI. The output emission spectra were plotted as histograms. Sub G1 populations are shown. Control values (medium alone) at 24, 48 and 72 hours were compared to those obtained at zero time and were found not to differ significantly from control values. AICAR-treated values were compared to the relevant controls. * = p < 0.05, ** = < 0.01; n=3 experiments, each carried out in triplicate

## Discussion

We have previously demonstrated that the genotype of cells (seemingly to be of the same type of cancer) may dictate the responses to targeted mediators of signal transduction being manipulated (Mooney et al., 2002; El-Masry et al., 2012; El-Masry et al., 2015). This taken together with the existence of crosstalk mechanisms in signal transduction that we (Quilliam et al., 1985; Dobson et al., 1990; El-Masry et al., 2015) and many others have shown highlights the complexity of these pathways. AMPK has a considerable profile as a target for treatment of cancer, especially of the breast. We have shown it to crosstalk with PI3K (El-Masry et al., 2015) which itself was subject to differences in action in terms of the three human breast cancer lines investigated here. In agreement with these observations, it has also been reported that Thalidezine-activated AMPK can sensitize apoptotic-resistant cancer cell lines and induce autophagic cell death in these cells, including MCF-7 breast cancer cells (Law et al., 2017). In addition, the anti-cancer effect of AICAR-activated AMPK was also reported in gastric cancer being able to activate the promoter of tumor suppressor genes through retinoid-related orphan receptor-α, an action which triggers apoptosis (Wang et al., 2017). We utilise cell lines as models, but even within specific cell lines there are variations and departures from genotypes. This is likely to approximate to the occurrence of cancers *in situ* at a cellular level. 

In the present study, we demonstrate further diversity of one major target for cancer therapies, AMPK, in terms of how it acts in cells having different inherent capacities. 

This work expands the current knowledge of potential modes of actions and the underlying mechanisms of response or resistance. Activation of AMPK in MCF-7, MDA-MB-231 and T47D cells provided a clue to the ability of AMPK to trigger apoptosis in all three cell lines regardless of p53 status, though T47D cells were less responsive to this signal (Figures 1, 2 and 3). We have previously shown that apoptotic signals were generated in these cell types at the mitochondrial level (El-Masry et al., 2012). In the present study we show that activation of AMPK resulted in translocation of cytosolic Bax to the outer mitochondrial membrane, which could be the cause of triggering mitochondrial apoptosis. Bax translocation was increased by prolonged incubation with AICAR, except in MCF-7 cells (Figure 2); this might have contributed to the acquired resistance of this cell line to the anti-proliferative effect of AICAR at high concentrations reported earlier (El-Masry et al., 2012). In addition, 3Bromopyruvate-activated AMPK resulted in increasing the expression of Bax in MCF-7 and caspase 3 in MDA-MB-231 cells to support TRAIL-stimulated apoptotic mechanisms (Chen et al., 2018). According to another study (Okoshi et al., 2008), phosphorylation of Ser46 was also associated with AMPK-mediated apoptosis in response to energy stresses, which suggests that the presence of functional p53 is required for the pro-apoptotic influence of AMPK. In the present study, irreversible inhibition of caspase 9 (the main mediator of mitochondrial apoptosis) abolished the anti-proliferative action of AICAR significantly in MCF-7 and MDA-MB-231 cells (Figure 3 A and B). The use of selective inhibitors for caspase 3 and 6 confirmed that the AICAR-induced apoptosis was mediated by caspase 3 and 6 in the MDA-MB-231 cell line, whereas only caspase 6 was involved in MCF-7 cells as they express non-functional truncated forms of caspase 3 (Riffell et al., 2011). This, however, did not affect their response to the pro-apoptotic effect of AMPK activation. This was in concordance with Riffell and colleagues (Riffell et al., 2011), who have reported the inability of truncated caspase 3 to protect MCF-7 cells from apoptosis. The inability of either caspase inhibitor to block the anti-proliferative effect of AICAR to a significant extent in T47D cells (Figure 3 C) confirms the lower apoptotic response in this cell line; this was also supported by the pattern of PARP cleavage (Figure 1 C), which took place in MCF-7 and MDA-MB-231 cells upon incubation with AICAR and was less clear in T47D cells. PARP cleavage was observed in HT-29 colon cancer cells in response to AMPK activation (Hwang et al., 2007; Khanal et al., 2011), which was in line with our finding. It has also been reported, however, that Metformin elicited AMPK-induced PARP cleavage in MDA-MB-231, but not in MCF-7 cells, an action that was dependent on the status of LKB1 (upstream kinase of AMPK) (Liu et al., 2009), which, to some extent, contradicts our results presented here. In addition, we have previously reported that staurosporine-mediated PARP cleavage in MCF-7 cells was greater and quicker than enzyme fragmentation in T47D cells (Mooney et al., 2002). These results taken together are in agreement with a recent study, wherein it is reported that activation of AMPK by N,N’-diarylurea FND-4b can trigger apoptosis in estrogen receptor-positive as well as triple negative breast cancer cell lines (Johnson et al., 2019). 

Analysis of cell cycle progression revealed that the anti-proliferative effect of AICAR in MCF-7 cells resulted from the ability of AICAR to increase the level of total p21 protein significantly (Figure 4). This was followed by an apoptotic response after 72 hours of incubation with AICAR, which seems to be *p53*-mediated (Figure 5). An explanation of this delayed apoptotic response in MCF-7 cells was also provided from a report (Abedin et al., 2007) that indicated an association between autophagy and a delayed apoptotic response in this cell line. This seems consistent with the lack of enhancement of Bax translocation upon prolonged stimulation with AICAR for 72 hours (Figure 2) and suggests a role of mitochondrial autophagy, which, perhaps, provides some protection at high concentrations of AICAR. The increased sensitivity to the anti-proliferative effects of AICAR observed at high concentrations in MDA-MB-231 cells was also confirmed by cell cycle analysis (Figure 5). A significant increase in apoptosis took place in this cell type upon prolonged incubation with AICAR (Figure 5). Although AICAR treatment induced a remarkable increase in p21 levels in this cell line when compared with the corresponding control (Figure 4 A), it was clear that the levels of p21 were less than those in the other two cell types. This might have been responsible for the greater pro-apoptotic influence of AICAR in MDA-MB-231 cells. This was also consistent with the reported apoptotic influence of AMPK activation in MDA-MB-231 cells, which was mediated by inhibition of Akt/FOXM1 signalling (Lee et al., 2018). Cell cycle analysis in T47D cells showed that AICAR-activated AMPK affected cell proliferation by induction of both cell cycle arrest and apoptosis (Figure 5). It was also obvious that these T47D cells have high levels of p21 (Figure 4), which might favour cell cycle arrest. This also suggests that the response of these cells to apoptosis is subtle and needs to be thoroughly examined. 

In conclusion, the cellular responses to the pro-apoptotic signals triggered by P-AMPK were greatly influenced by the cellular genetics of the cells involved. AICAR administration induced a remarkable elevation of total p21 levels regardless of *p53* status. Low levels of this regulator in MDA-MB-231 cells also favor an apoptotic response to AICAR. Therefore, AICAR-activated AMPK produced anti-proliferative effects by concomitant regulation of cell cycle progression and induction of apoptosis, and these modes of actions were influenced by the genetic makeup of the cells involved. 

The importance of this report is to indicate that a new awareness of cancer mechanisms may translate to better and more tolerable approaches to the treatment of cancers that could become available to patients. Biochemical signalling mechanisms demonstrate obvious targets for cancer therapy and some impetus is already being felt in respect of these in terms of cancer treatment. This came about from the knowledge that oncogenic targets (e.g. overexpression of key enzymes) are usually present in these pathways in cancer. They also crosstalk, which places another level of regulation. Here, we used established clonal lines of the same cancer type to elucidate that very distinctive biochemical differences exist between them, although technically they are all breast cancers. The aim is to demonstrate that therapies based on signalling mechanisms, rather than by the more traditional and randomised DNA approach of chemotherapy, are not only likely to be better targeted and less invasive (due to the robustness and interactive nature of these biochemical pathways), but allow that genetic traits may be addressed directly. In essence, the basis of better treatment for cancer sufferers is substantially possible in terms of patient specific therapy that could ultimately be determined by the evidence presented in biopsy material. 
